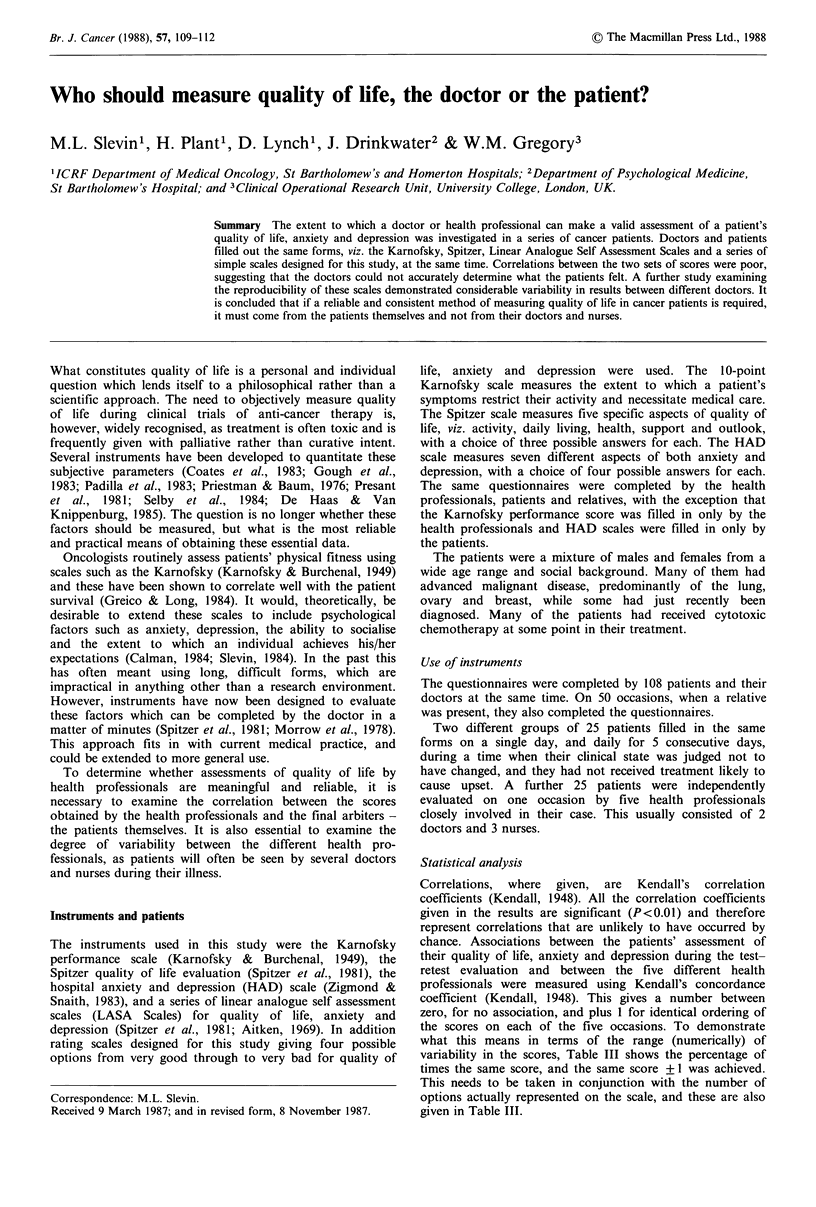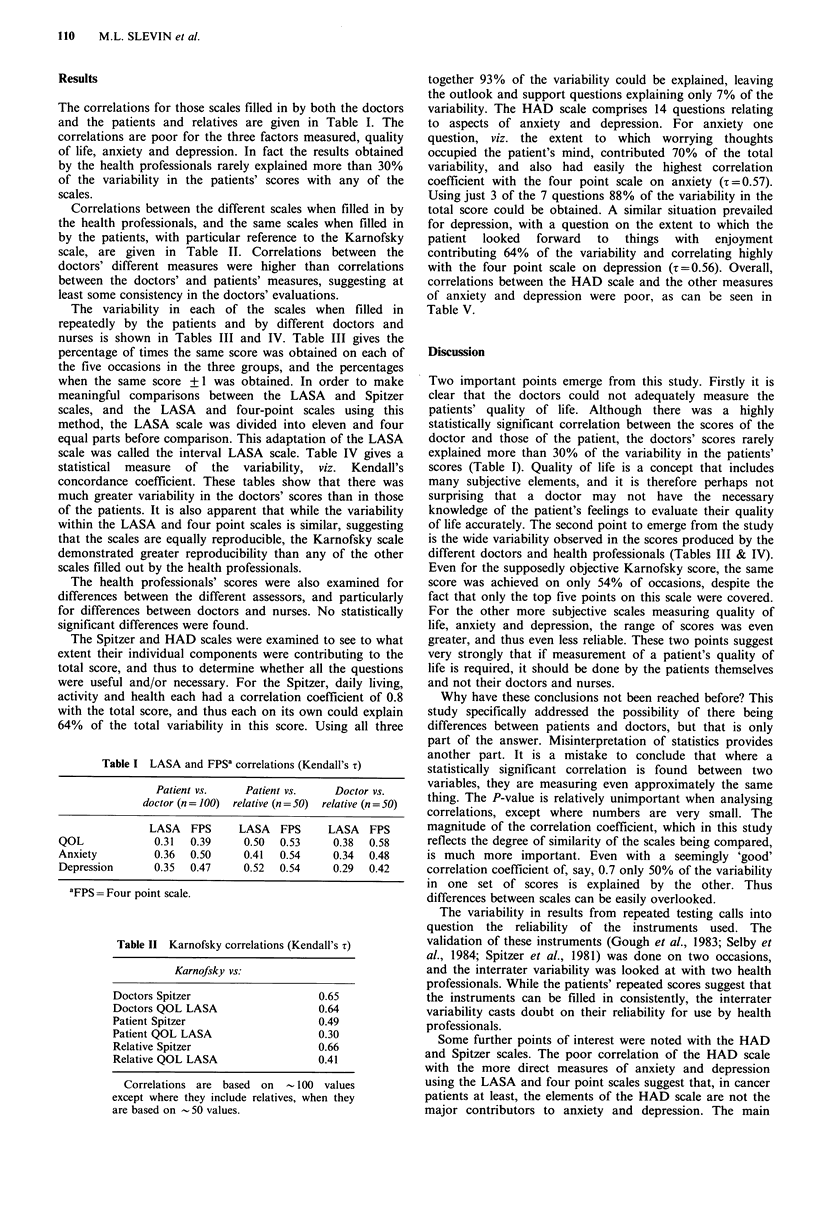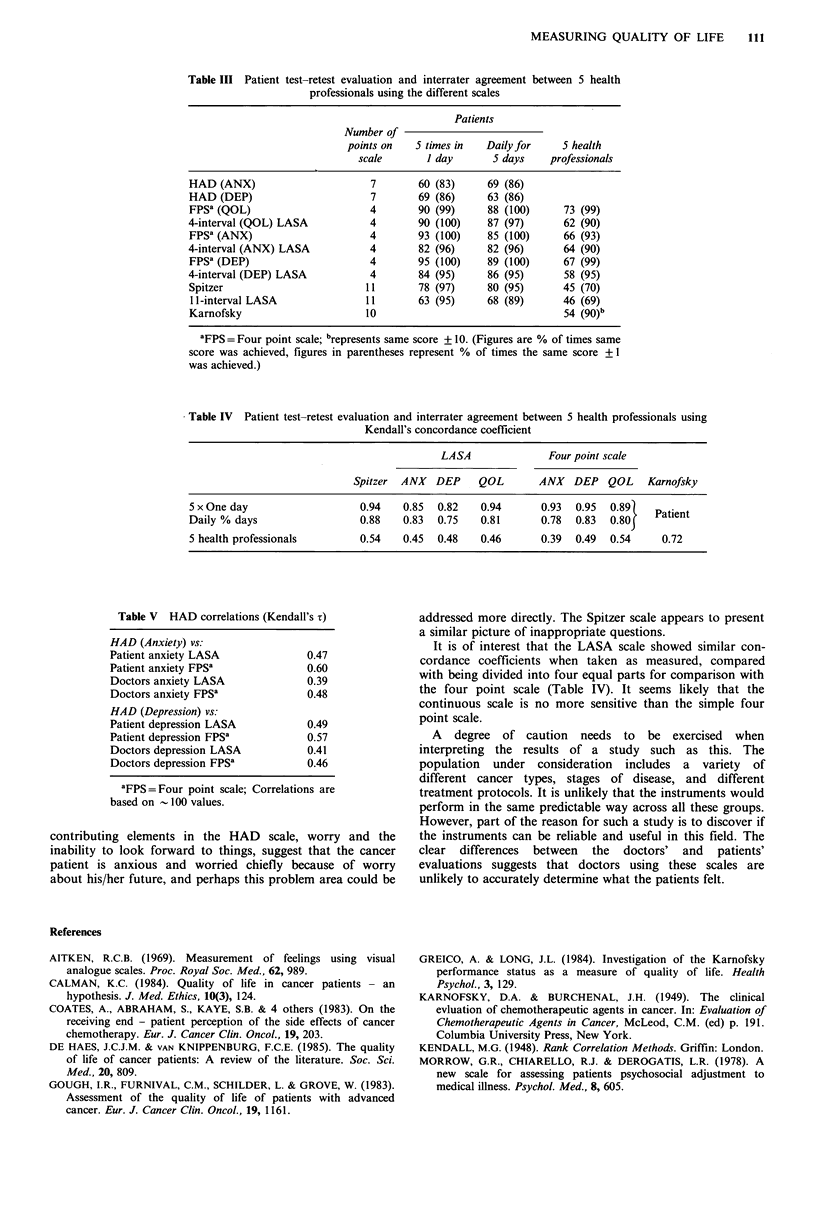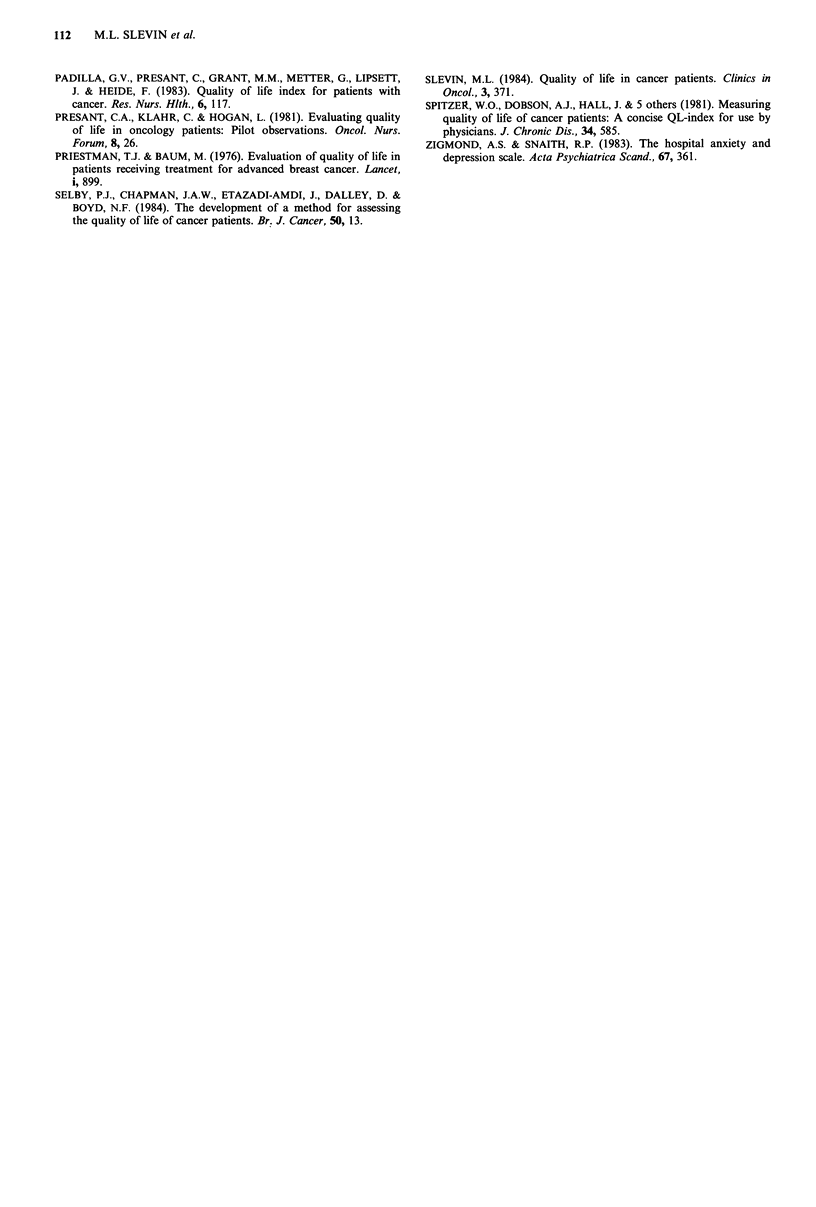# Who should measure quality of life, the doctor or the patient?

**DOI:** 10.1038/bjc.1988.20

**Published:** 1988-01

**Authors:** M. L. Slevin, H. Plant, D. Lynch, J. Drinkwater, W. M. Gregory

**Affiliations:** ICRF Department of Medical Oncology, St Bartholomew's Hospital, London, UK.

## Abstract

The extent to which a doctor or health professional can make a valid assessment of a patient's quality of life, anxiety and depression was investigated in a series of cancer patients. Doctors and patients filled out the same forms, viz. the Karnofsky, Spitzer, Linear Analogue Self Assessment Scales and a series of simple scales designed for this study, at the same time. Correlations between the two sets of scores were poor, suggesting that the doctors could not accurately determine what the patients felt. A further study examining the reproducibility of these scales demonstrated considerable variability in results between different doctors. It is concluded that if a reliable and consistent method of measuring quality of life in cancer patients is required, it must come from the patients themselves and not from their doctors and nurses.


Br. J. Cancer (1988), 57, 109-112 © The Macmillan Press Ltd., 1988

### Who should measure quality of life, the doctor or the patient?

M.L. Slevin[1], H. Plant[1], D. Lynch[1], J. Drinkwater[2] & W.M. Gregory[3]

[1]*ICRF Department of Medical Oncology, St Bartholomew's and Homerton Hospitals;* [2]*Department of Psychological Medicine, St Bartholomew's Hospital; and* [3]*Clinical Operational Research Unit, University College, London, UK.*

**Summary** The extent to which a doctor or health professional can make a valid assessment of a patient's quality of life, anxiety and depression was investigated in a series of cancer patients. Doctors and patients filled out the same forms, *viz.* the Karnofsky, Spitzer, Linear Analogue Self Assessment Scales and a series of simple scales designed for this study, at the same time. Correlations between the two sets of scores were poor, suggesting that the doctors could not accurately determine what the patients felt. A further study examining the reproducibility of these scales demonstrated considerable variability in results between different doctors. It is concluded that if a reliable and consistent method of measuring quality of life in cancer patients is required, it must come from the patients themselves and not from their doctors and nurses.

What constitutes quality of life is a personal and individual question which lends itself to a philosophical rather than a scientific approach. The need to objectively measure quality of life during clinical trials of anti-cancer therapy is, however, widely recognised, as treatment is often toxic and is frequently given with palliative rather than curative intent. Several instruments have been developed to quantitate these subjective parameters (Coates *et al.*, 1983; Gough *et al.*, 1983; Padilla *et al.*, 1983; Priestman & Baum, 1976; Presant *et al.*, 1981; Selby *et al.*, 1984; De Haas & Van Knippenburg, 1985). The question is no longer whether these factors should be measured, but what is the most reliable and practical means of obtaining these essential data.

Oncologists routinely assess patients' physical fitness using scales such as the Karnofsky (Karnofsky & Burchenal, 1949) and these have been shown to correlate well with the patient survival (Greico & Long, 1984). It would, theoretically, be desirable to extend these scales to include psychological factors such as anxiety, depression, the ability to socialise and the extent to which an individual achieves his/her expectations (Calman, 1984; Slevin, 1984). In the past this has often meant using long, difficult forms, which are impractical in anything other than a research environment. However, instruments have now been designed to evaluate these factors which can be completed by the doctor in a matter of minutes (Spitzer *et al.*, 1981; Morrow *et al.*, 1978). This approach fits in with current medical practice, and could be extended to more general use.

To determine whether assessments of quality of life by health professionals are meaningful and reliable, it is necessary to examine the correlation between the scores obtained by the health professionals and the final arbiters – the patients themselves. It is also essential to examine the degree of variability between the different health professionals, as patients will often be seen by several doctors and nurses during their illness.

### Instruments and patients

The instruments used in this study were the Karnofsky performance scale (Karnofsky & Burchenal, 1949), the Spitzer quality of life evaluation (Spitzer *et al.*, 1981), the hospital anxiety and depression (HAD) scale (Zigmond & Snaith, 1983), and a series of linear analogue self assessment scales (LASA Scales) for quality of life, anxiety and depression (Spitzer *et al.*, 1981; Aitken, 1969). In addition rating scales designed for this study giving four possible options from very good through to very bad for quality of life, anxiety and depression were used. The 10-point Karnofsky scale measures the extent to which a patient's symptoms restrict their activity and necessitate medical care. The Spitzer scale measures five specific aspects of quality of life, *viz.* activity, daily living, health, support and outlook, with a choice of three possible answers for each. The HAD scale measures seven different aspects of both anxiety and depression, with a choice of four possible answers for each. The same questionnaires were completed by the health professionals, patients and relatives, with the exception that the Karnofsky performance score was filled in only by the health professionals and HAD scales were filled in only by the patients.

The patients were a mixture of males and females from a wide age range and social background. Many of them had advanced malignant disease, predominantly of the lung, ovary and breast, while some had just recently been diagnosed. Many of the patients had received cytotoxic chemotherapy at some point in their treatment.

### *Use of instruments*

The questionnaires were completed by 108 patients and their doctors at the same time. On 50 occasions, when a relative was present, they also completed the questionnaires.

Two different groups of 25 patients filled in the same forms on a single day, and daily for 5 consecutive days, during a time when their clinical state was judged not to have changed, and they had not received treatment likely to cause upset. A further 25 patients were independently evaluated on one occasion by five health professionals closely involved in their case. This usually consisted of 2 doctors and 3 nurses.

### *Statistical analysis*

Correlations, where given, are Kendall's correlation coefficients (Kendall, 1948). All the correlation coefficients given in the results are significant ($P<0.01$) and therefore represent correlations that are unlikely to have occurred by chance. Associations between the patients' assessment of their quality of life, anxiety and depression during the test–retest evaluation and between the five different health professionals were measured using Kendall's concordance coefficient (Kendall, 1948). This gives a number between zero, for no association, and plus 1 for identical ordering of the scores on each of the five occasions. To demonstrate what this means in terms of the range (numerically) of variability in the scores, Table III shows the percentage of times the same score, and the same score $\pm 1$ was achieved. This needs to be taken in conjunction with the number of options actually represented on the scale, and these are also given in Table III.

Correspondence: M.L. Slevin.
Received 9 March 1987; and in revised form, 8 November 1987.

### Results

The correlations for those scales filled in by both the doctors and the patients and relatives are given in Table I. The correlations are poor for the three factors measured, quality of life, anxiety and depression. In fact the results obtained by the health professionals rarely explained more than 30% of the variability in the patients' scores with any of the scales.

Correlations between the different scales when filled in by the health professionals, and the same scales when filled in by the patients, with particular reference to the Karnofsky scale, are given in Table II. Correlations between the doctors' different measures were higher than correlations between the doctors' and patients' measures, suggesting at least some consistency in the doctors' evaluations.

The variability in each of the scales when filled in repeatedly by the patients and by different doctors and nurses is shown in Tables III and IV. Table III gives the percentage of times the same score was obtained on each of the five occasions in the three groups, and the percentages when the same score $\pm 1$ was obtained. In order to make meaningful comparisons between the LASA and Spitzer scales, and the LASA and four-point scales using this method, the LASA scale was divided into eleven and four equal parts before comparison. This adaptation of the LASA scale was called the interval LASA scale. Table IV gives a statistical measure of the variability, *viz.* Kendall's concordance coefficient. These tables show that there was much greater variability in the doctors' scores than in those of the patients. It is also apparent that while the variability within the LASA and four point scales is similar, suggesting that the scales are equally reproducible, the Karnofsky scale demonstrated greater reproducibility than any of the other scales filled out by the health professionals.

The health professionals' scores were also examined for differences between the different assessors, and particularly for differences between doctors and nurses. No statistically significant differences were found.

The Spitzer and HAD scales were examined to see to what extent their individual components were contributing to the total score, and thus to determine whether all the questions were useful and/or necessary. For the Spitzer, daily living, activity and health each had a correlation coefficient of 0.8 with the total score, and thus each on its own could explain 64% of the total variability in this score. Using all three together 93% of the variability could be explained, leaving the outlook and support questions explaining only 7% of the variability. The HAD scale comprises 14 questions relating to aspects of anxiety and depression. For anxiety one question, *viz.* the extent to which worrying thoughts occupied the patient's mind, contributed 70% of the total variability, and also had easily the highest correlation coefficient with the four point scale on anxiety ($\tau = 0.57$). Using just 3 of the 7 questions 88% of the variability in the total score could be obtained. A similar situation prevailed for depression, with a question on the extent to which the patient looked forward to things with enjoyment contributing 64% of the variability and correlating highly with the four point scale on depression ($\tau = 0.56$). Overall, correlations between the HAD scale and the other measures of anxiety and depression were poor, as can be seen in Table V.

**Table I** LASA and FPS[a] correlations (Kendall's $\tau$)

| | *Patient vs. doctor (n = 100)* | | *Patient vs. relative (n = 50)* | | *Doctor vs. relative (n = 50)* | |
|---|---|---|---|---|---|---|
| | LASA | FPS | LASA | FPS | LASA | FPS |
| QOL | 0.31 | 0.39 | 0.50 | 0.53 | 0.38 | 0.58 |
| Anxiety | 0.36 | 0.50 | 0.41 | 0.54 | 0.34 | 0.48 |
| Depression | 0.35 | 0.47 | 0.52 | 0.54 | 0.29 | 0.42 |

[a]FPS = Four point scale.

**Table II** Karnofsky correlations (Kendall's $\tau$)

| *Karnofsky vs:* | |
|---|---|
| Doctors Spitzer | 0.65 |
| Doctors QOL LASA | 0.64 |
| Patient Spitzer | 0.49 |
| Patient QOL LASA | 0.30 |
| Relative Spitzer | 0.66 |
| Relative QOL LASA | 0.41 |

Correlations are based on $\sim$100 values except where they include relatives, when they are based on $\sim$50 values.

### Discussion

Two important points emerge from this study. Firstly it is clear that the doctors could not adequately measure the patients' quality of life. Although there was a highly statistically significant correlation between the scores of the doctor and those of the patient, the doctors' scores rarely explained more than 30% of the variability in the patients' scores (Table I). Quality of life is a concept that includes many subjective elements, and it is therefore perhaps not surprising that a doctor may not have the necessary knowledge of the patient's feelings to evaluate their quality of life accurately. The second point to emerge from the study is the wide variability observed in the scores produced by the different doctors and health professionals (Tables III & IV). Even for the supposedly objective Karnofsky score, the same score was achieved on only 54% of occasions, despite the fact that only the top five points on this scale were covered. For the other more subjective scales measuring quality of life, anxiety and depression, the range of scores was even greater, and thus even less reliable. These two points suggest very strongly that if measurement of a patient's quality of life is required, it should be done by the patients themselves and not their doctors and nurses.

Why have these conclusions not been reached before? This study specifically addressed the possibility of there being differences between patients and doctors, but that is only part of the answer. Misinterpretation of statistics provides another part. It is a mistake to conclude that where a statistically significant correlation is found between two variables, they are measuring even approximately the same thing. The *P*-value is relatively unimportant when analysing correlations, except where numbers are very small. The magnitude of the correlation coefficient, which in this study reflects the degree of similarity of the scales being compared, is much more important. Even with a seemingly 'good' correlation coefficient of, say, 0.7 only 50% of the variability in one set of scores is explained by the other. Thus differences between scales can be easily overlooked.

The variability in results from repeated testing calls into question the reliability of the instruments used. The validation of these instruments (Gough *et al.*, 1983; Selby *et al.*, 1984; Spitzer *et al.*, 1981) was done on two occasions, and the interrater variability was looked at with two health professionals. While the patients' repeated scores suggest that the instruments can be filled in consistently, the interrater variability casts doubt on their reliability for use by health professionals.

Some further points of interest were noted with the HAD and Spitzer scales. The poor correlation of the HAD scale with the more direct measures of anxiety and depression using the LASA and four point scales suggest that, in cancer patients at least, the elements of the HAD scale are not the major contributors to anxiety and depression. The main

**Table III** Patient test–retest evaluation and interrater agreement between 5 health professionals using the different scales

| | Number of points on scale | Patients: 5 times in 1 day | Patients: Daily for 5 days | 5 health professionals |
|---|---|---|---|---|
| HAD (ANX) | 7 | 60 (83) | 69 (86) | |
| HAD (DEP) | 7 | 69 (86) | 63 (86) | |
| FPS[a] (QOL) | 4 | 90 (99) | 88 (100) | 73 (99) |
| 4-interval (QOL) LASA | 4 | 90 (100) | 87 (97) | 62 (90) |
| FPS[a] (ANX) | 4 | 93 (100) | 85 (100) | 66 (93) |
| 4-interval (ANX) LASA | 4 | 82 (96) | 82 (96) | 64 (90) |
| FPS[a] (DEP) | 4 | 95 (100) | 89 (100) | 67 (99) |
| 4-interval (DEP) LASA | 4 | 84 (95) | 86 (95) | 58 (95) |
| Spitzer | 11 | 78 (97) | 80 (95) | 45 (70) |
| 11-interval LASA | 11 | 63 (95) | 68 (89) | 46 (69) |
| Karnofsky | 10 | | | 54 (90)[b] |

[a]FPS = Four point scale; [b]represents same score ±10. (Figures are % of times same score was achieved, figures in parentheses represent % of times the same score ±1 was achieved.)

**Table IV** Patient test–retest evaluation and interrater agreement between 5 health professionals using Kendall's concordance coefficient

| | Spitzer | LASA: ANX | LASA: DEP | LASA: QOL | Four point scale: ANX | Four point scale: DEP | Four point scale: QOL | Karnofsky |
|---|---|---|---|---|---|---|---|---|
| 5 × One day | 0.94 | 0.85 | 0.82 | 0.94 | 0.93 | 0.95 | 0.89 | Patient |
| Daily % days | 0.88 | 0.83 | 0.75 | 0.81 | 0.78 | 0.83 | 0.80 | |
| 5 health professionals | 0.54 | 0.45 | 0.48 | 0.46 | 0.39 | 0.49 | 0.54 | 0.72 |

**Table V** HAD correlations (Kendall's $\tau$)

| | |
|---|---|
| *HAD (Anxiety) vs:* | |
| Patient anxiety LASA | 0.47 |
| Patient anxiety FPS[a] | 0.60 |
| Doctors anxiety LASA | 0.39 |
| Doctors anxiety FPS[a] | 0.48 |
| *HAD (Depression) vs:* | |
| Patient depression LASA | 0.49 |
| Patient depression FPS[a] | 0.57 |
| Doctors depression LASA | 0.41 |
| Doctors depression FPS[a] | 0.46 |

[a]FPS = Four point scale; Correlations are based on ~100 values.

contributing elements in the HAD scale, worry and the inability to look forward to things, suggest that the cancer patient is anxious and worried chiefly because of worry about his/her future, and perhaps this problem area could be addressed more directly. The Spitzer scale appears to present a similar picture of inappropriate questions.

It is of interest that the LASA scale showed similar concordance coefficients when taken as measured, compared with being divided into four equal parts for comparison with the four point scale (Table IV). It seems likely that the continuous scale is no more sensitive than the simple four point scale.

A degree of caution needs to be exercised when interpreting the results of a study such as this. The population under consideration includes a variety of different cancer types, stages of disease, and different treatment protocols. It is unlikely that the instruments would perform in the same predictable way across all these groups. However, part of the reason for such a study is to discover if the instruments can be reliable and useful in this field. The clear differences between the doctors' and patients' evaluations suggests that doctors using these scales are unlikely to accurately determine what the patients felt.

### References

AITKEN, R.C.B. (1969). Measurement of feelings using visual analogue scales. *Proc. Royal Soc. Med.*, **62**, 989.

CALMAN, K.C. (1984). Quality of life in cancer patients – an hypothesis. *J. Med. Ethics*, **10(3)**, 124.

COATES, A., ABRAHAM, S., KAYE, S.B. & 4 others (1983). On the receiving end – patient perception of the side effects of cancer chemotherapy. *Eur. J. Cancer Clin. Oncol.*, **19**, 203.

DE HAES, J.C.J.M. & VAN KNIPPENBURG, F.C.E. (1985). The quality of life of cancer patients: A review of the literature. *Soc. Sci. Med.*, **20**, 809.

GOUGH, I.R., FURNIVAL, C.M., SCHILDER, L. & GROVE, W. (1983). Assessment of the quality of life of patients with advanced cancer. *Eur. J. Cancer Clin. Oncol.*, **19**, 1161.

GREICO, A. & LONG, J.L. (1984). Investigation of the Karnofsky performance status as a measure of quality of life. *Health Psychol.*, **3**, 129.

KARNOFSKY, D.A. & BURCHENAL, J.H. (1949). The clinical evluation of chemotherapeutic agents in cancer. In: *Evaluation of Chemotherapeutic Agents in Cancer*, McLeod, C.M. (ed) p. 191. Columbia University Press, New York.

KENDALL, M.G. (1948). *Rank Correlation Methods*. Griffin: London.

MORROW, G.R., CHIARELLO, R.J. & DEROGATIS, L.R. (1978). A new scale for assessing patients psychosocial adjustment to medical illness. *Psychol. Med.*, **8**, 605.

PADILLA, G.V., PRESANT, C., GRANT, M.M., METTER, G., LIPSETT, J. & HEIDE, F. (1983). Quality of life index for patients with cancer. *Res. Nurs. Hlth.*, **6**, 117.

PRESANT, C.A., KLAHR, C. & HOGAN, L. (1981). Evaluating quality of life in oncology patients: Pilot observations. *Oncol. Nurs. Forum*, **8**, 26.

PRIESTMAN, T.J. & BAUM, M. (1976). Evaluation of quality of life in patients receiving treatment for advanced breast cancer. *Lancet*, **i**, 899.

SELBY, P.J., CHAPMAN, J.A.W., ETAZADI-AMDI, J., DALLEY, D. & BOYD, N.F. (1984). The development of a method for assessing the quality of life of cancer patients. *Br. J. Cancer*, **50**, 13.

SLEVIN, M.L. (1984). Quality of life in cancer patients. *Clinics in Oncol.*, **3**, 371.

SPITZER, W.O., DOBSON, A.J., HALL, J. & 5 others (1981). Measuring quality of life of cancer patients: A concise QL-index for use by physicians. *J. Chronic Dis.*, **34**, 585.

ZIGMOND, A.S. & SNAITH, R.P. (1983). The hospital anxiety and depression scale. *Acta Psychiatrica Scand.*, **67**, 361.